# Implementation of Stereotactic MRI-Guided Adaptive Radiotherapy (SMART) for Hepatobiliary and Pancreatic Cancers in the United Kingdom – Fifty in Five

**DOI:** 10.7759/cureus.15075

**Published:** 2021-05-17

**Authors:** Andrew Gaya, Philip Camilleri, Adam Nash, Donna Hughes, James Good

**Affiliations:** 1 Clinical Oncologist, GenesisCare, London, GBR; 2 Radiotherapy, Oxford University Hospitals NHS Foundation Trust, Oxford, GBR; 3 Radiotherapy, GenesisCare, Oxford, GBR; 4 Radiotherapy, Queen Elizabeth Hospital, Birmingham, GBR

**Keywords:** stereotactic radiotherapy, liver metastases, pancreatic cancer, mridian®, mr-linac, hepatobiliary tumours

## Abstract

The first MRIdian® MR linear accelerator (MR-Linac; ViewRay, Oakwood Village, Ohio) in the United Kingdom went live in December 2019 following a record installation time. Stereotactic MRI-guided Adaptive Radiotherapy (SMART) has since been implemented and has advantages of excellent soft tissue definition of both target and organs at risk (OARs), real-time target and OAR visualisation on cine-MRI, daily recontouring of target and critical OARs with live online plan adaptation/re-optimisation, and automatic respiratory-gated treatment delivery. We present a multi-disciplinary narrative and technical description of how this innovative technique was implemented for hepatobiliary (HPB) cancers. In particular, we explain how a collaborative approach and desire to push the boundaries and improve outcomes enabled 50 patients to be treated in the first five months, many with technically challenging tumours not always deliverable on other platforms. Physics, dosimetry, radiographer, and clinician perspectives on implementing SMART are presented. MRIdian® single fraction lung stereotactic ablative radiotherapy (SABR) will shortly be implemented along with innovative research in conjunction with our academic partners.

## Introduction

MR linear accelerator (MR-Linac) technology holds great promise for patients with tumours in challenging anatomical locations, such as the liver, pancreas and upper abdomen, via a paradigm shift in image guidance combined with an online adaptation of target volumes and organs at risk (OARs) and a re-optimisation of treatment plans. There are currently two operational systems - Elekta’s Unity® (Elekta, Stockholm, Sweden) and ViewRay’s MRIdian® (ViewRay, Oakwood Village, Ohio) [1]. Whilst they are used for conventional radiotherapy or stereotactic ablative radiotherapy (SABR), we believe the greatest therapeutic yield derives from the latter and have used MRIdian® solely as a SABR platform.

MRIdian provides continuous real-time MR imaging with superior soft tissue definition compared with cone-beam CT, facilitating precise setup, and on-table treatment plan adaptation. Treatment delivery is automatically breath-hold gated so the dose is only delivered when positioning is optimal, removing the need for an internal target volume (ITV), reducing the planning target volume (PTV) margin and volume of normal tissue irradiated. This may mitigate radiation-induced toxicities, and the need for invasive fiducial markers is avoided. Benefits are likely to be greatest in areas of significant inter and/or intrafraction motion of target or OARs [2-3].

European Society of Medical Oncology (ESMO) guidelines [4] specify a role for SABR in oligometastatic disease originating from non-small-cell lung cancer (NSCLC), colorectum cancer, melanoma, renal cancer, and sarcoma. The recently published long-term outcomes of Stereotactic Ablative Radiotherapy for the Comprehensive Treatment of Oligometastases (SABR-COMET) [5] highlight SABR’s growing role in the multimodality treatment of oligometastases; five-year overall survival (OS) was 42% in the SABR arm vs 17% with standard of care alone.

For decades, surgical resection and adjuvant chemotherapy/radiotherapy have been the cornerstone of primary and secondary HPB cancer treatment [6]. These patients pose challenges on many fronts: less than 20% are resectable at diagnosis, not all patients are fit for surgery or chemotherapy and when radiotherapy is employed, dose escalation is often challenging with conventional techniques due to the location of targets and proximity of dose-limiting OARs. Heterogeneous SABR dose distributions partly mitigate these challenges, especially for inoperable patients or those with unresectable pancreatic tumours [7]. Dose escalation and integration of SABR with systemic therapies are the mainstays of clinical trials. Results are promising, with published data showing median OS up to 19.7 months for SABR plus chemotherapy, with acceptable toxicity [8].

Large retrospective cohorts have shown improvement in OS and local control for SABR-delivered biologically effective doses (BED) >= 100Gy_10_ [9]. MRI-guided radiotherapy has emerged as a promising modality to achieve accurate delivery of escalated doses for oligometastatic or unresectable primary HPB tumours [10]. A retrospective multi-institutional analysis of 44 patients with locally advanced pancreatic cancer treated on MRIdian® demonstrated that dose-escalated SABR can improve OS compared with standard doses. The median follow-up was 17 months. At two years, 49% of the high-dose group was alive, compared with 30% of the standard dose group (p=0.03). On multi-variate analysis, radiation dose and duration of induction chemotherapy correlated with OS [11].

Liver tumours up to 3 cm can be treated with surgery or local ablative therapies, including radiofrequency ablation (RFA) and SABR with similar outcomes [12-13]. Historically, the conventional radiotherapeutic approach to larger (>3 cm) and/or unresectable tumours has been limited by concerns regarding organ motion and radiation-induced liver disease (RILD) with many patients being treated only with systemic agents. Some SABR studies have yielded good results with large HPB tumours, with one-year local control exceeding 90% and acceptable toxicity [14]. Twenty-six patients with HPB tumours (six hepatocellular cancers (HCC), two cholangiocarcinomas, and 18 liver metastases) treated on MRIdian® were analysed in a retrospective multi-institutional study. The median dose was 50 Gy in 10 fractions and the median PTV was 98.2 cc (range 13-2034 cc). With a median follow-up of 21.2 months while one-year and two-year survival were 69% and 60%, respectively, with only 2% grade 3 toxicity. Freedom from local progression at the median follow-up was 80.4%. Progression-free survival (PFS) in the entire cohort was 35% [15].

With a vision to bring this technology to the UK and work with partners to develop the evidence base, the UK’s first MRIdian® system went live in December 2019. Major challenges were faced by the team at every stage from procurement, through installation, commissioning and training to deliver the UK’s first stereotactic MRI-guided adaptive radiotherapy (SMART) for HPB cancers. This article describes that process, and the lessons learned.

## Technical report

A worldwide network of MRIdian® centres has been treating patients for over five years. Prior, the fastest Viewray installation timeline was 53 days. The accelerated installation timeline agreed was 45 days. The site works started in May 2019 and the construction of a modular high-density bunker, shielded door and radiofrequency cage in June 2019. Space was at a premium due to site layout and existing facilities. Existing services needed to be rerouted away from the new facility. MRIdian® can be accommodated in a smaller bunker than alternative systems, and this was a factor in choosing the system.

**Figure 1:**
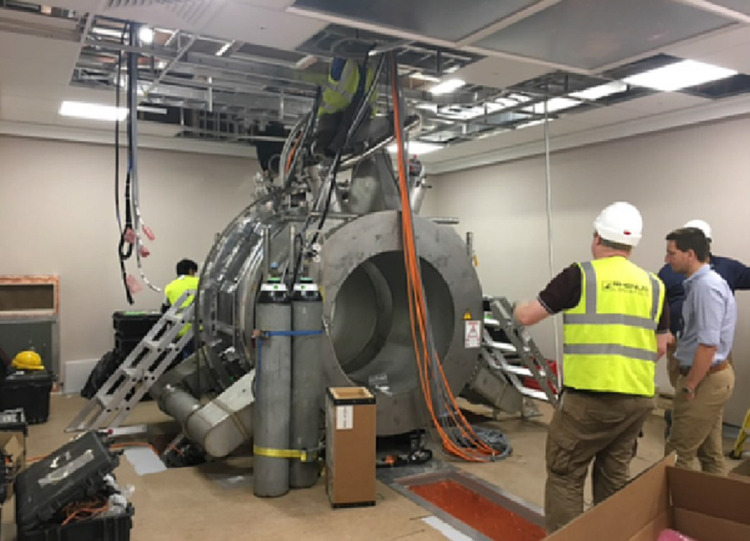
Installation of the MRIDian bore

Delivery of components was in September 2019; the magnetic field was ramped up on October 14 and radiation beam-on one week later (Figure 1). The acceptance test procedure (ATP) for the imaging system was started on October 23 followed by radiation beam ATP. We took possession of the MRIdian® on November 7, 2019, 45 days after the start of installation.

Commissioning and verification were scheduled for three weeks. MRIdian® is a standalone treatment platform consisting of a 6 MV flattening filter-free (FFF) beam with a dose rate of 600 MU/min and a double-stacked 0.5 cm multileaf collimator (MLC). The hardware and treatment planning system (TPS) are incorporated into a single platform using a central patient database with no connection to an external record and verify (R&V) system. The system is supplied with a generic beam model in the TPS, and the linac is matched to this. Physics commissioning is primarily validating this beam model for different clinical scenarios, and validation of complex treatment plan dose delivery with the addition of beam gating and tumour tracking using dynamic phantoms and MR-compatible measurement devices.

The minimal perturbation of delivered dose distribution, whilst still offering excellent image quality, is an advantage of the low strength magnetic field in the MRIdian®. The perturbation of dose distribution (through the electron return effect) from the magnetic field must be assessed. Whilst the effect can be observed in a 0.35T field, its effect is minimal compared to 1.5T. This can be modelled and accounted for during treatment plan optimisation on the MRIdian® TPS.

The ATP for both imaging and radiation could be improved to streamline the commissioning process. Connecting the MR-Linac to an R&V system is a priority for data tracking and easy transfer of patients in a central network environment. Maintaining close working relationships with the vendor is essential.

Bringing the team together

GenesisCare has a network of 190 centres across four countries and there is usually a site that has already implemented a new treatment platform. With MRIdian®, there was no such precedent. Also because this was the first machine in the UK, there were no local peers, collaborative hospitals or oncologists to call on. That is the reality of innovation: isolating, and it requires organisations and individuals to operate outside of their comfort zones.

It was recognised from the start that involving innovative and committed oncologists would be central to the project’s success. Unlike the NHS or our centres in Australia or Spain, the UK had not hitherto employed doctors but instead had a national network of over 150 independent oncologists. Recruiting new oncologists to part-time positions was an early step. The eight doctors chosen all held NHS employment when approached. Each had to negotiate job plan changes to liberate time, and all had to take a leap of faith in taking on a new way of working.

The 2019 ViewRay User Meeting provided the starting point for consultant training. A visit to Siteman Cancer Centre (Washington University, St Louis) cemented the clinicians’ commitment and enthusiasm; seeing the MRIdian® safely delivering treatment not seen before in the UK generated energy, optimism and enthusiasm that fuelled the next 11 months. Other meetings the same year provided good reflection points and a chance to reconnect with newly acquired collaborators and colleagues.

The wider MRIdian® team were fortunate to be able to visit the Miami Cancer Institute, Heidelberg University Hospital, and the Carbone Cancer Centre, University of Wisconsin, throughout 2019, in training for “go live”. This made it possible for the team to gain the requisite knowledge in a short timeline.

For the operational team, a comprehensive training schedule was developed. This included shadowing clinical staff at Henry Ford Cancer Center; on-site training included weekly webinars with applications specialists on treatment planning and delivery; use of simulated treatment software allowing experience to be gained with the online adaptive process; role-specific training courses were delivered by ViewRay. The team welcomed international experts to host interactive workshops and share expertise, including MRI radiology learning sessions. In Autumn 2019, credentialing was completed with final visits to VUmc Amsterdam and Acibadem Maslak Hospital, Istanbul.

The first patient began their five-fraction ultra-hypofractionated prostate treatment on December 9, 2019, just 80 days after installation began with experienced radiographers from VUmc and applications specialists from ViewRay. The biggest success was taking eight oncologists and providing world-class training to build a team to deliver truly innovative world-class radiotherapy.

Physics perspective

The ViewRay MRIdian® combines a 0.35T split superconducting magnet with a 6 MV linear accelerator [16]. This results in a linac with significantly different characteristics from a conventional linac, as summarised in Table 1. As a result, many of the standard tests used in commissioning are not relevant and new measuring techniques are required. Moreover, due to factors including the MR environment, limited bore size and lack of radiation light field, equipment used during commissioning and quality control (QC) may not be compatible with the MR-Linac.

**Table 1:** Characteristics of the ViewRay MRIdian compared to a conventional linac treatment platform SAD: source-axis distance; MLC: multileaf collimator

ViewRay MRIdian	Conventional Linac
No flattening filter	Has a flattening filter
Single 6 MV energy	Multiple energies
Beam up to 27 x 24 cm2	Beam up to 40 x 40 cm^2^
90 cm SAD	100 cm SAD
No light field	Has light field
Virtual isocentre lasers	Fixed lasers
Magnetic field	No magnetic field
Double stack, double-focused MLCs	Standard MLC leaves
No collimator rotation	Rotating collimator
Integrated treatment planning system	Independent treatment planning system

There is currently a lack of MRI expertise within the radiotherapy physics community, for example, only a small number of centres have experience with dedicated MR-sims [17]. Engagement with experts in this field is key in terms of commissioning and MR safety training.

The introduction of any new system, especially one as groundbreaking as MRIdian®, includes a learning curve [18] where expertise is gained in how to problem-solve errors or develop solutions for edge cases. Working closely with the ViewRay technical and engineering support has enabled high machine up-time. Ensuring a consistent approach and standards of quality assurance (QA) across different technologies has required re-thinking processes, for example, accounting for previous radiotherapy treatment. There are several aspirations for service development such as incorporating a synthetic CT system so that only an MRI planning scan is required for most patients. There are currently no synthetic CT solutions designed to work with the MRIdian® and, therefore, collaboration with vendors is required.

Dosimetrist perspective

MRIdian® fundamentally changes the role of the dosimetrist in the treatment pathway. For most Linac-based treatments, the same plan will be delivered at each fraction. Thus, the planning process is based on internal anatomy visualised at simulation, with no scope to change or re-optimise based on inter- or intrafraction organ motion. The opportunity to escalate dose is limited by the need to account for changes in OAR position from fraction to fraction.

Conversely, plan adaptation is at the heart of each MRIdian® treatment. SMART requires the use of both optimisation structures and planning objectives to carry out daily adaptation effectively and quickly [19]. The optimisation volumes can be complex and difficult to implement effectively because of the need to re-populate volumes based on changes to target and OARs. One of the key requirements at planning is to define a set of margin expansions and Boolean operations, which will produce the required optimisation structures. On completion of planning, testing is carried out to ensure that the plans are optimisable during treatment. Complexity is increased with abdominal/thorax plans where ablative doses are required directly adjacent to radio-sensitive OARs which by their nature are prone to significant interfraction motion.

**Figure 2:**
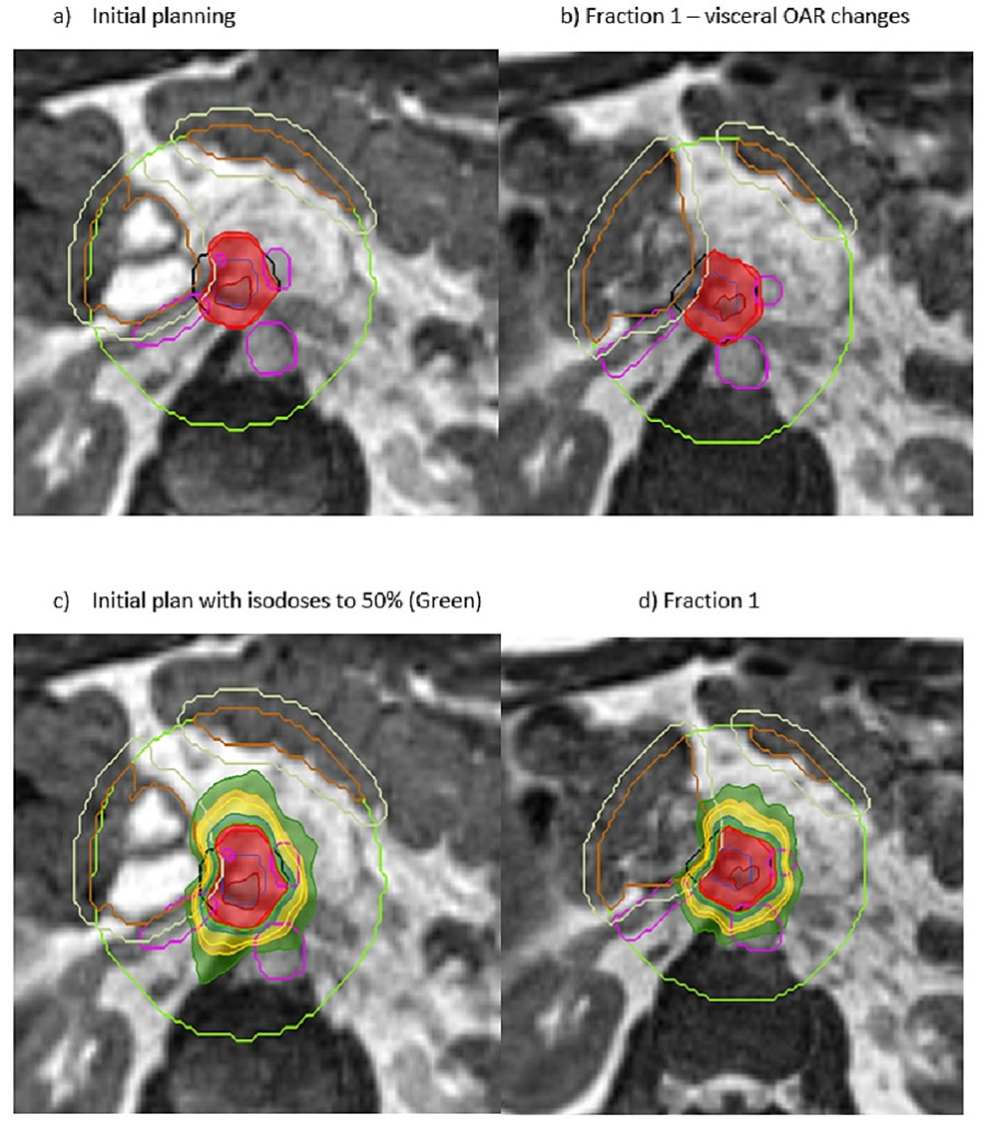
Cholangiocarcinoma OAR Overlap Cholangiocarcinoma OAR overlap (PTVhigh) volume with 100% isodose line conforming to PTVhigh (PTV-Visceral OAR+5 mm and Vessels) 130% hot spot in the GTV. brown = visceral OAR; purple = blood vessels; cream = visceral OAR + margin; black = PTV; red = PTVprescribe; green = 3 cm ring structure around PTV OAR: organ at risk; PTV: planning target volume; GTV: gross tumour volume

**Figure 3:**
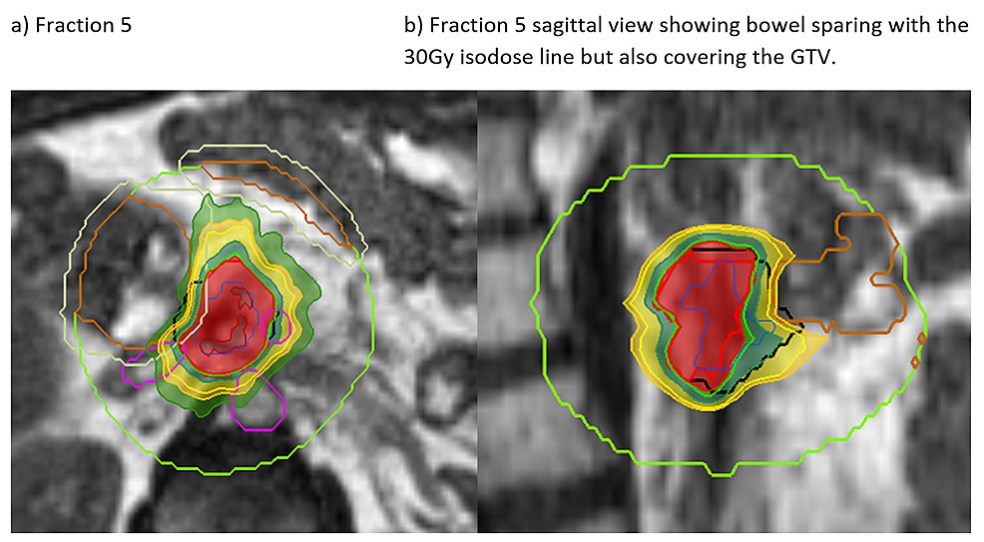
Cholangiocarcinoma OAR and PTV Cholangiocarcinoma OAR overlap (PTVhigh) volume with 100% isodose line conforming to PTVhigh (PTV-VisceralOAR+5 mm and Vessels) 130% hot spot in the GTV. brown = visceral OAR; purple = blood vessels; cream = visceral OAR + margin; black = PTV; red = PTVprescribe; green = 3 cm ring structure around PTV OAR: organ at risk; PTV: planning target volume; GTV: gross tumour volume

**Figure 4:**
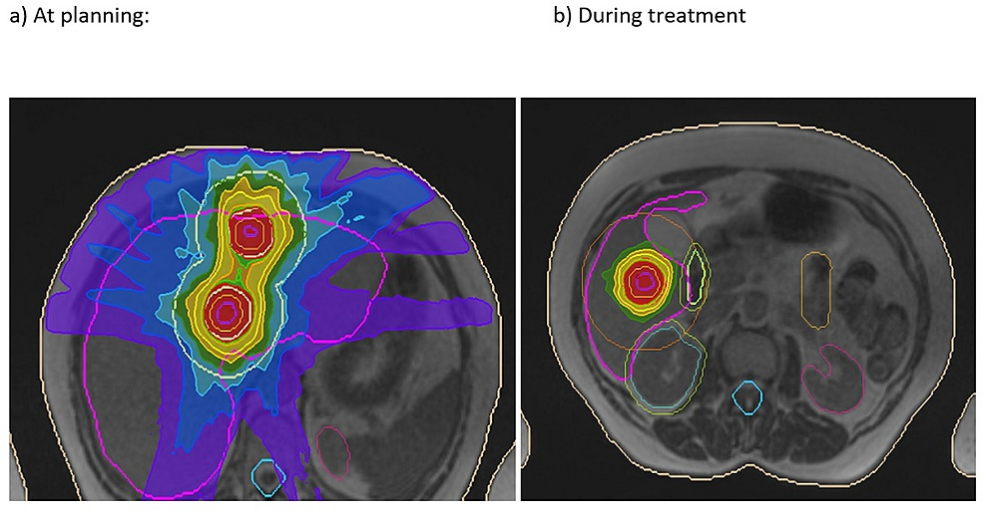
Treatment of Three Liver Metastases The two superior metastases and inferior met. The inferior volume shows down to the 50% isodose and the local OARs (a) at the time of planning and (b) at the time of treatment. See also Figure 5 OAR: organ at risk

**Figure 5:**
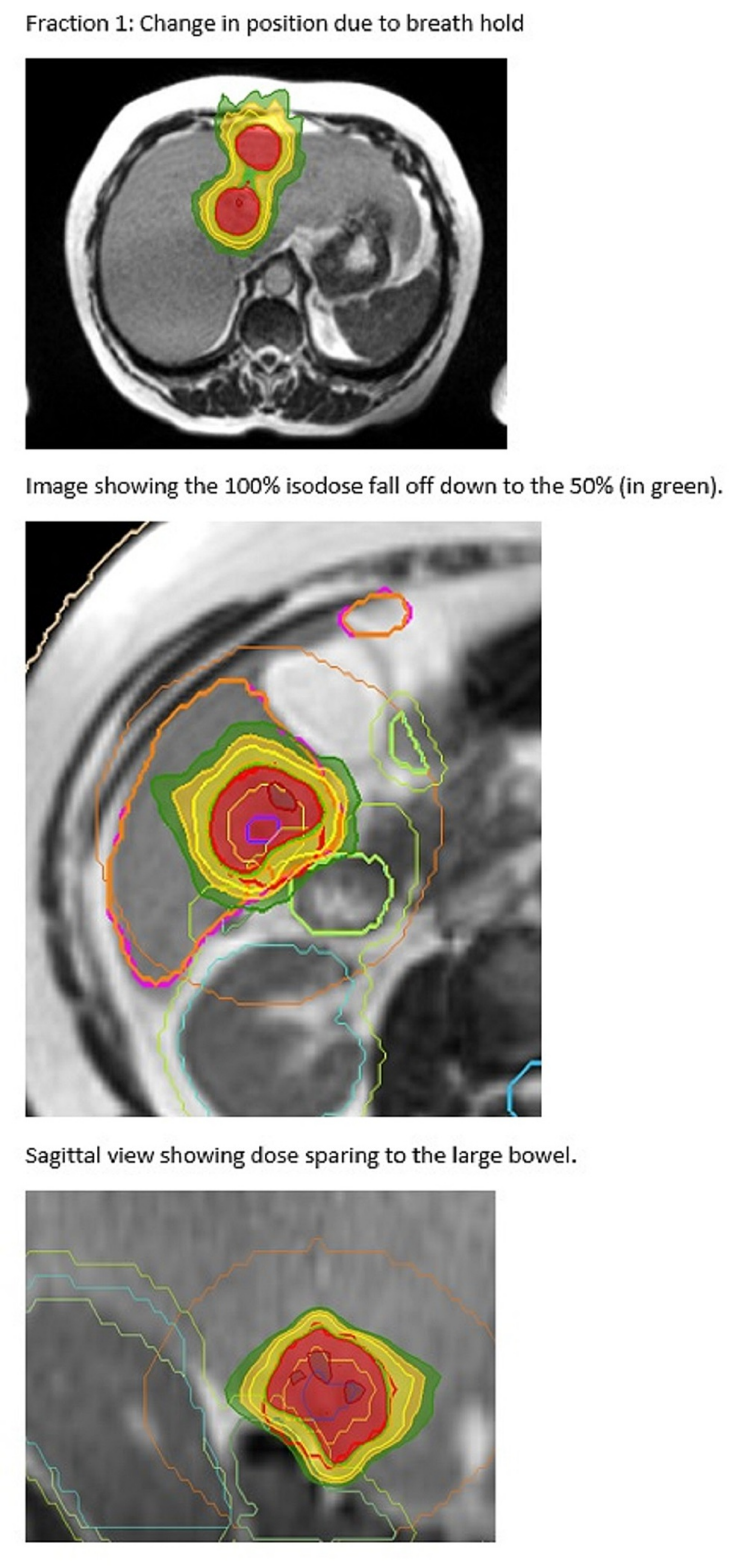
Three Liver Metastases Treatment Plan Two superior volumes and an inferior volume. Illustrates the change in position with breath-hold and variation in the position of OARs during treatment (compared to at the time of planning in Figure 4).

**Figure 6:**
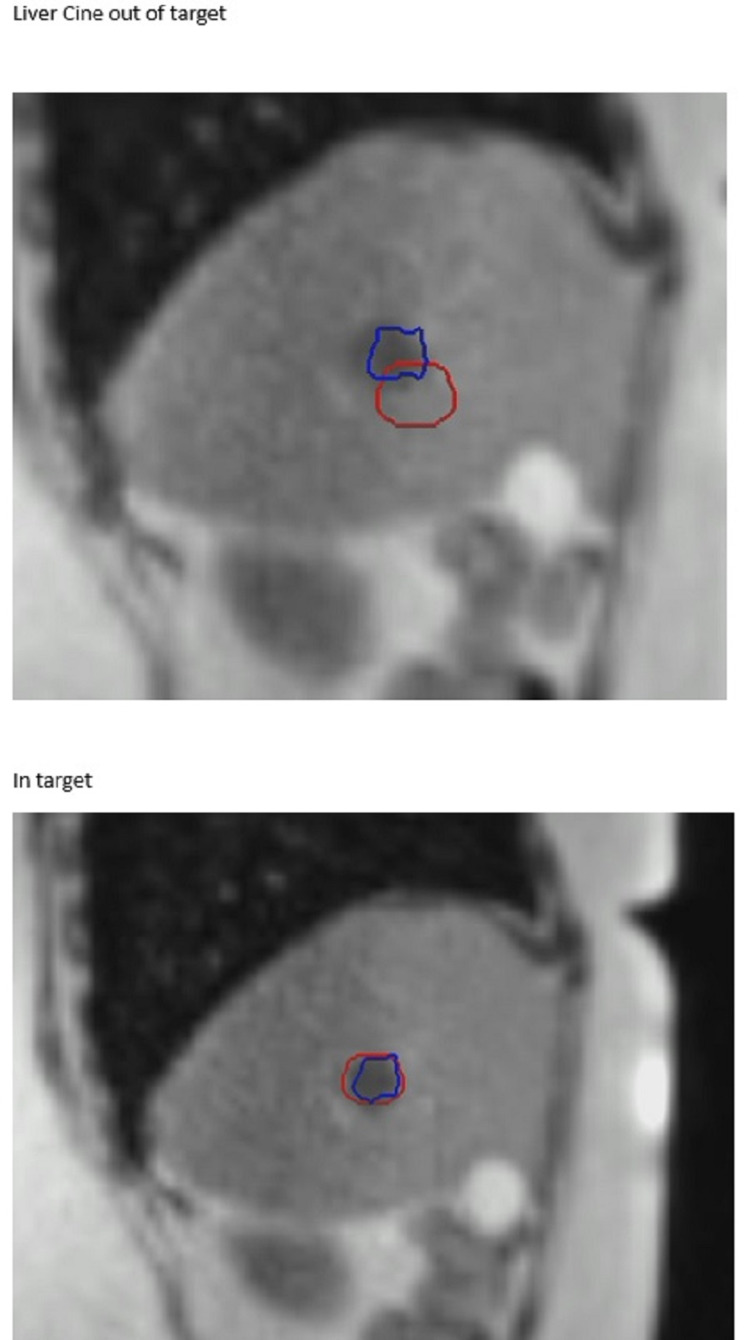
Three Liver Metastases Treatment - Cine View Images Tumour outlined in blue, with the breath-hold target in red. The 0.35T image is grainy at four frames per second, but the Primovist contrast enables accurate delineation and tracking of the target.

Planning treatments specifically to be adapted is a complex task, as over-optimised plans can be difficult or impossible to re-plan quickly whilst the patient is on-table. This requires a different attitude when compared to standard planning concepts of multiple iterations and fine balancing of planning parameters. The range of cases provides challenges and rewards, with pancreas, liver, nodal and reirradiation SABR being much of the complex workload (Figures 2-6). All target volumes, OARs and treatment plans are peer-reviewed.

Radiographer perspective

For MRIDian® radiographers, the way of working has been turned upside down. We now form part of a dynamic, multi-disciplinary team. In a standard radiotherapy workflow, a patient will receive a treatment planning CT one to two weeks before the start of treatment. During this time, several steps are carried out by a team of dosimetrists, physicists, doctors and radiographers to produce a treatment plan ready for the first fraction.

**Figure 7:**
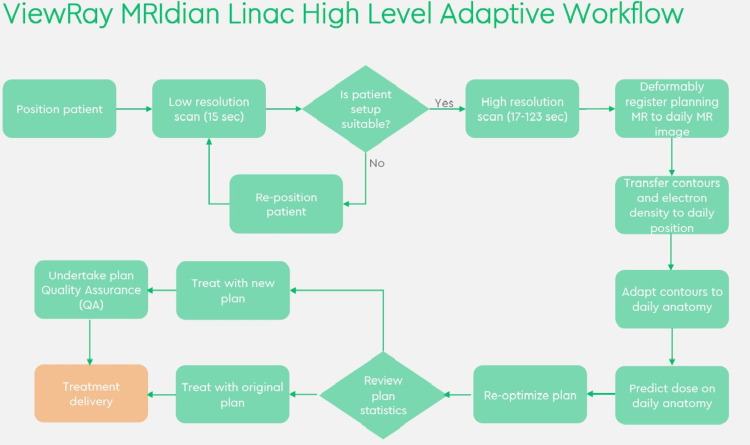
MRIDian Adaptive Workflow

As part of the on-table adaptive workflow (Figure 7), this process must be reduced from days to minutes. To achieve this, close inter-disciplinary working between radiographers, dosimetrists, physicists and doctors is required. The need to undertake several complex tasks during each adaptive treatment also increases the time for each fraction to over one hour. Each discipline seamlessly steps in and out of the workflow at various points, often discussing their thoughts and actions as they work; the MR-Linac control area is a melting pot of expertise, knowledge and ideas.

One of the most crucial relationships has been with diagnostic MRI colleagues. Not only do they provide a robust MR safety framework under which to develop the service, but also regularly act as a sounding board around implant safety.

Beam-gated breath-hold deliveries are routinely performed for abdomen or chest treatment. Patients view a monitor at the rear of the MRIdian® where they visualise their tumour on a cine-MRI image and direct their own breath-hold to perfectly position their tumour within a region of interest (ROI) boundary, which triggers the beam on. Alternatively, radiographers can verbally coach them.

Whilst this advanced technology is exciting to work with, patient care skills remain at the core of a radiographer’s role. Almost 10% of our patients have been claustrophobic and rarely believe that they will tolerate lying in the bore for up to 90 minutes; with compassionate verbal coaching and feedback, no treatment fraction has yet been abandoned for this reason.

HPB oncologist perspective

Being the first UK centre to implement SMART for HPB cancers has been challenging and required a multidisciplinary collaborative approach, allied with significant input and assistance from colleagues at other MRIdian® centres. With the increased confidence given by the soft tissue definition of the MRIdian®, recontouring of targets and OARs and daily plan re-optimisation, and automatic respiratory gating with live cine-MRI, it has been possible to dose escalate primary pancreatic cancer from 33 Gy in five fractions to 50 Gy in five fractions [19]. This has been achieved with all OARs in tolerance and to date, no acute >grade 3 toxicity has been observed. Patient-reported outcome measurement (PROM) data has been collected on all patients. We have reduced planning margins and thus the volume of normal tissue irradiated, with no need for ITV definition and a PTV margin of 3 mm. The most common toxicities have been fatigue and nausea. Our outcome and toxicity data will be published in due course.

Other “firsts” have been the delivery of retreatment radiotherapy for pancreatic cancer to a dose of 40 Gy in five fractions after the previous 54 Gy. Several patients with multiple liver metastases (Figures 4-6) and upper abdominal multifocal nodal recurrences have been treated. Primovist® liver-specific contrast enables outstanding visualisation of tumours, which the delivery system can track. When treating multiple targets simultaneously, one is selected for live tracking and the position of the others is monitored in relation to this. Difficult-to-visualise OARs on CT such as the boundary between the medial duodenal wall and pancreas tumour or the bile ducts are delineated without issue on the MR-Linac.

The image quality is excellent using small fields of view, with refinements to come in future software upgrades. Magnetic field strength is only one factor in determining image quality, with less distortion and planning uncertainty at 0.35T [20]. Significant interfraction and intrafraction motion with critical OARs such as the stomach, small bowel and duodenum have been noted, necessitating almost complete recontouring at times. For intrafraction motion, the beam is gated off whilst the target is outside PTV. We note that standard planning margins on a pre-treatment cone-beam computed tomography (CBCT) are not sufficient to prevent a partial geographic miss of the target during delivery of some fractions.

It is a novel and rewarding experience working in “real-time” on set, side by side with radiographers, physics and dosimetry and learning the intricacies of patient positioning, image matching and treatment delivery. There is always time pressure because the patient is on the table, and we try to keep treatment times reasonable. Patient set-up, positioning, imaging, recontouring, replanning and gated treatment delivery can take 90-120 mins in some challenging cases.

Non-HPB oncologist perspective

For uro oncologists, the radiological anatomy of the pelvis is not an issue, and many will maintain a working knowledge of abdominal anatomy. However, adapting cases involving liver and pancreas tumours with their surrounding OARs requires a suitable accreditation process.

This included a specific radiological anatomy training day where consultants buddied up with clinicians of different site-specialisations and reviewed MRIdian® scans of both the abdomen and pelvis with radiologist supervision. This was accompanied by didactic teaching and contouring practice.

In the run-up to going live, we practised building confidence and developing good teamwork. This included talking through the difficult aspects of specific anatomy and sharing useful tips such as utilising all plane views (coronal, sagittal and axial) when contouring the small bowel or duodenum to ensure the anatomic path of the organ is correct. Our pre-coronavirus disease (COVID) rota allowed for two clinicians to be present on set (upper gastrointestinal (GI) and urological) to provide support and advice.

The adapting clinician does not take decisions regarding major amendments of the gross tumour volume (GTV). There are usually minor adjustments required to ensure the daily target position is covered by the referring clinician’s contour. A handover document allows the oncologists to communicate any subtleties and changes made. Practice is important for adapting abdominal OARs, as is the utilisation of the pre-existing plan and support from colleagues and radiographers. The superior soft tissue definition of MRI certainly helps.

Cases treated to date

As of April 2021, we completed SABR treatment on almost 200 patients (Table 2), delivering over 1000 fractions, each one individually recontoured with plan adaptation and re-optimisation and with gated treatment delivery if appropriate. Dose and fractionation are illustrated in Table 3. Three hundred patients are anticipated to be treated in the next year.

**Table 2:** Patients Treated on UK MRIdian

Patients Treated on UK MRIdian April 2021	
Lung (primary or met)	8
Lymph nodes	30
Liver (HCC + mets)	29
Pelvis reirradiation	9
Pancreas	41
Prostate	71
Over 1000 fractions now delivered on 197 patients	

**Table 3:** Dose and Fractionation Used in Our First 200 Patients

	Prostate	Liver	Pancreas	Lung	Lymph Nodes	Renal
30Gy in 5F	2		1	1	17	
30Gy in 3F		3		1	1	
35Gy in 5F			1		2	
36Gy in 3F		1				2
36.25Gy in 5F	90					
40Gy in 3F					2	
40Gy in 5F		7	38	1	9	1
45Gy in 3F		2				
45Gy in 5F			2			
50Gy in 5F		14	3		2	
50Gy in 8F						
55Gy in 5F		3		2		
60Gy in 5F		3				
60Gy in 8F				2		
TOTAL	92	33	45	7	33	3

Research and development

A number of contemporary trends in radiation oncology converge in the growing role of MR-Linac. They facilitate the safe and accurate delivery of SABR to a variety of anatomical targets, especially those that are difficult to treat using CBCT guidance and a non-adaptive treatment pathway. The trend towards hypofractionation will only be accelerated by the necessity to adapt treatment paradigms to the possibility that COVID-19 will be endemic for some time. In addition, the ablation of metastatic disease is likely to grow in prominence. Should trials investigating the role of SABR in polymetastatic disease (such as SABR-COMET-10) and in oligoprogression prove positive, treatment techniques that minimise the amount of normal tissue irradiated are likely to boost the feasibility and safety of these approaches. The possibility of integrating on-table functional imaging data into target volume delineation and dose prescription decisions offers the possibility of personalising treatment using both anatomical and biological information in one seamless workflow.

The capital and operational costs of MR-Linac are significant. A coordinated programme of research and service evaluation that transcends barriers between institutions will be required if the oncology community in the UK is to develop evidence for the feasibility and clinical value necessary to convince NHS commissioners. This programme will need to take into account the different commissioning environments in each of the devolved administrations and strive to ensure that patients across the country have equality of access if clinical benefit is demonstrated. For example, SABR for a limited oligometastatic disease has now been routinely commissioned in England but SABR for localised pancreatic cancer has not. Our first prospective research initiative will therefore be a three-way partnership with the University of Oxford and a charitable partner to characterise clinical outcomes and PROMs following SMART in this patient group. Further cohorts in other clinical situations will follow.

## Discussion

The first MRIdian® MR linear accelerator (MR-Linac) in the United Kingdom went live following a record installation time. SMART has the advantages of excellent soft tissue definition of both the target and organs at risk (OAR), real-time target and OAR visualisation on cine-MRI, daily adaptive recontouring of target and critical OARs with live online plan adaptation and re-optimisation and automatic respiratory-gated treatment delivery. The soft tissue contrast definition is particularly notable with HPB tumours and central lung tumours. We also note major benefits for reirradiation patients in having the confidence of being able to determine accurate OAR dose on a daily basis and re-optimise the plan. In addition, the machine can account for intrafraction motion, which gives a further level of confidence. The TruFisp MRI sequence, which is approximately 60% T1 weighted, 40% T2-weighted, provides excellent image contrast for malignant pathology. Primovist, with at least a one-hour delay from the injection, provides excellent visualisation of liver tumours. The latest software upgrades on the MRIdian® also allow for conventional T1 and T2 images and diffusion-weighted imaging. Cine imaging has also been upgraded to eight frames per second. Future developments may include artificial intelligence (AI)-aided recontouring. All these advances improve the speed of the workflow. Prostates can be treated in 45 minutes, and complex HPB in 60-90 minutes using the adaptive workflow.

Toxicity has been very manageable. For HPB tumours, in particular, the main toxicities have been fatigue (largely grade 1) and nausea (grades 1-2). Nausea has been reduced significantly since we introduced a practice of administering prophylactic ondansetron 4 mg BID throughout treatment. One patient had a GI bleed following treatment (grade 3). There have been no grade 4 or 5 toxicities to date. The prostate cohort has also tolerated the treatment very well with largely grade 1-2 lower urinary tract symptoms. A significant proportion of patients with nodal disease have reported no toxicity whatsoever. All toxicity is captured via an electronic PROMS database, which all patients complete.

Operating during the COVID-19 pandemic has been challenging. We saw an increase in referrals for SABR largely due to cutbacks in surgical services through the height of the pandemic in order to release intensive care unit capacity and ventilators. For prostate and pancreatic patients, in particular, a five-fraction SABR protocol was favourable in terms of reducing the number of hospital visits. We also saw increased referrals for oligometastatic disease to delay systemic therapy, and hope the improved outcomes for patients will encourage the future increase in volumes of patients with both oligometastatic and oligoprogressive disease.

A collaborative approach and desire to push the boundaries and improve outcomes enabled 50 patients to be treated in the first five months, and over 200 in the first year, many with technically challenging tumours not always treatable on other platforms. Physics, dosimetry, radiographers and clinicians working together seamlessly allow for this to happen.

## Conclusions

The MR-Linac in Oxford is the UK’s first MRIdian® system. We have successfully utilised SMART for the treatment of HPB tumours and abdominal reirradiation to push the boundaries of what is possible. Changes to oncologists’ job plans for allowing machine time, functional multidisciplinary team working and collaboration are essential to run a successful service. We will continue research initiatives in conjunction with our partners at Oxford University and look forward to networking with other sites in the UK and internationally.
